# The Effects of Functional Magnetic Stimulation on Rectus Abdominis Muscle Size and Abdominal Subcutaneous Adipose Tissue Thickness

**DOI:** 10.7759/cureus.96057

**Published:** 2025-11-04

**Authors:** Roberto Valdivia

**Affiliations:** 1 Aesthetic Medicine, Association of Longevity and Aesthetic Medicine, Escazú, CRI; 2 Aesthetic Medicine, Dr. Valdivia Sing Aesthetic and Anti-aging Medicine, Escazú, CRI

**Keywords:** aesthetic medicine, body shaping, functional magnetic stimulation, muscle hypertrophy, waist circumference

## Abstract

Background

Functional magnetic stimulation (FMS) is a non-invasive technology that induces supramaximal muscle contractions through rapidly changing magnetic fields. It has been proposed as a method to enhance muscle hypertrophy and reduce localized adiposity, but objective clinical evidence remains limited. This pilot case series implemented a systematic intervention and measurement protocol to evaluate the effects of FMS on rectus abdominis muscle thickness and abdominal subcutaneous adipose tissue.

Methods

Ten healthy adults (six female, four male; mean age 32.7 years) underwent 10 sessions of abdominal FMS over three weeks using the Tesla Former® device (Iskra Medical, Otoče, Slovenia). Waist circumference and body weight were measured at baseline and at post-treatment. Participants also underwent abdominal magnetic resonance imaging (MRI) to assess rectus abdominis muscle thickness and subcutaneous adipose tissue thickness. Data was analyzed using IBM SPSS Statistics for Windows, version 28 (IBM Corp., Armonk, New York, United States).

Results

All participants completed the study with no loss to follow-up. Mean waist circumference decreased significantly by −2.76 ± 1.37 cm (*p* < 0.001), while mean body weight showed a non-significant reduction of −0.6 ± 1.35 kg (*p* > 0.05). MRI analysis demonstrated a significant increase in rectus abdominis thickness (+5.98 ± 1.87 mm, *p* < 0.001) and a significant decrease in subcutaneous adipose tissue thickness (−4.35 ± 2.80 mm, *p* < 0.001). All participants reported subjective improvement in abdominal strength and appearance.

Conclusion

FMS was associated with increased rectus abdominis muscle thickness, reduced abdominal subcutaneous adipose tissue, and decreased waist circumference in this pilot case series, with high patient satisfaction and no serious adverse events. These findings suggest that FMS may be a promising non-invasive option for abdominal shaping, but larger randomized controlled trials are necessary to confirm its efficacy and long-term benefits.

## Introduction

Body contouring and abdominal muscle strengthening are increasingly sought-after goals in both clinical and aesthetic medicine [1]. Traditional methods for improving abdominal muscle tone and reducing fat include lifestyle modification, exercise, and dietary interventions [2]. More invasive approaches, such as liposuction or abdominoplasty, can achieve noticeable results but are associated with surgical risks, cost, and downtime [3,4]. As a result, there is growing interest in non-invasive technologies that can enhance muscle mass while reducing subcutaneous adipose tissue [5].

Functional magnetic stimulation (FMS) is a relatively novel technique that uses high intensity, rapidly changing magnetic fields to induce supramaximal contractions of targeted muscle groups [6]. Unlike voluntary exercise, FMS can stimulate deeper muscle fibers and achieve contraction intensities that are difficult to replicate during conventional training [7]. Preliminary reports suggest that repetitive supramaximal contractions may promote muscle hypertrophy, improve core strength, and simultaneously influence local fat metabolism [8-10]. Despite these promising claims, the available evidence is limited, and objective data using imaging-based measurements remain scarce.

Given this background, we conducted a pilot case series to evaluate the effects of FMS on the rectus abdominis muscle and abdominal subcutaneous fat. Using magnetic resonance imaging (MRI) and standardized anthropometric measures, we aimed to assess changes in muscle thickness and subcutaneous adipose tissue following a series of abdominal FMS treatments. This exploratory study was designed to provide preliminary evidence on feasibility, safety, and potential efficacy, thereby laying the foundation for larger controlled trials. The primary objective of current study was to evaluate the effects of FMS on rectus abdominis muscle thickness and abdominal subcutaneous adipose tissue. The secondary objectives was to assess the effects of FMS on waist circumference and weight reduction. 

## Materials and methods

Study design

This pilot case series, conducted at Dr. Valdivia Sing Aesthetic and Anti-aging Medicine, Escazú, Costa Rica, employed a systematic intervention and measurement protocol to evaluate the effects of FMS on rectus abdominis muscle size and abdominal subcutaneous adipose tissue thickness. All participants underwent the same treatment protocol, and measurements were collected at baseline and at 10 weeks after the final treatment session.

Participants

Ten healthy adult volunteers were enrolled. Eligible participants were required to have a stable body weight, no history of abdominal surgery within the past year, and no contraindications to FMS therapy or MRI. Exclusion criteria included pregnancy, the presence of metallic implants or pacemakers, and ongoing structured weight-loss or body-contouring interventions. Participants were instructed to maintain their usual dietary and physical activity habits during the study period. Written informed consent was obtained from all individuals prior to enrolment.

Intervention

All participants underwent a standardized course of FMS consisting of 10 treatment sessions administered using the Tesla Former Prestige® device (Iskra Medical, Otoče, Slovenia). Treatments were delivered with two large FMS applicators positioned side by side over the abdomen and secured with adjustable straps to ensure consistent contact and positioning. Each session lasted 30 minutes and followed the preset “Abdomen 2” program, which includes a sequence of frequency modulations designed to activate different muscle fiber types. The stimulation intensity ranged from 2% to 100% of the device’s maximum magnetic field strength of 3 T and was individually adjusted at each session according to participant comfort and tolerability, aiming to maintain a strong yet tolerable muscle contraction. Sessions were conducted every other day, with a two-day interval between treatments, for a total of 10 sessions over approximately three weeks. Baseline assessments were performed prior to the first session, and post-intervention measurements were obtained at 10 weeks after the final treatment session. Participants were monitored throughout the study for any adverse events or discomfort during and after treatment.

Outcome measures

The primary outcomes were changes in rectus abdominis muscle thickness and abdominal subcutaneous adipose tissue thickness. Measurements were performed at baseline and 10 weeks following the final treatment.

Anthropometric Measurements

Waist circumference was measured at the midpoint between the iliac crest and the costal margin with participants in the standing position. Measurements were performed using a flexible, non-elastic tape measure. All participants were weighed at baseline and follow-up to check for changes in body weight.

MRI

Rectus abdominis muscle thickness and subcutaneous adipose tissue thickness were measured at the level of the umbilicus through abdominal MRI using standardized imaging protocols by a radiologist.

Patient-Reported Outcomes

Patient subjective satisfaction was recorded using a questionnaire (see Appendcies), which included questions about the satisfaction with muscle appearance, strength, and the perceived muscle contraction strength during muscle stimulation, which was evaluated on a 1-5 scale (1 = none; 2 = low; 3 = moderate, 4 = high, 5 = very high). The patients were also asked about their willingness to repeat and recommend the treatment to others.

Data analysis

Data were analyzed using IBM SPSS Statistics for Windows, version 28.0 (IBM Corp., Armonk, New York, United States). Descriptive statistics were used to summarize participant characteristics. The Shapiro-Wilk test was applied to test the normality of the data. As the data were normally distributed, paired t-tests were performed to compare pre- and post-treatment changes in muscle thickness, subcutaneous fat thickness, and waist circumference. A *p*-value of <0.05 was considered statistically significant.

Ethics

Ethical approval was obtained from the Ethics Committee of the Costa Rican Association of Longevity and Aesthetic Medicine (approval number: ACOLME/EC/2024/211). All procedures were conducted in accordance with the principles of the Declaration of Helsinki.

## Results

Ten participants (n = 10), with a mean age of 32.7 years, were included in this pilot case series. Six participants were female and four were male. All subjects completed the 10-session FMS protocol, and no participants were lost to follow-up.

**Figure 1:**
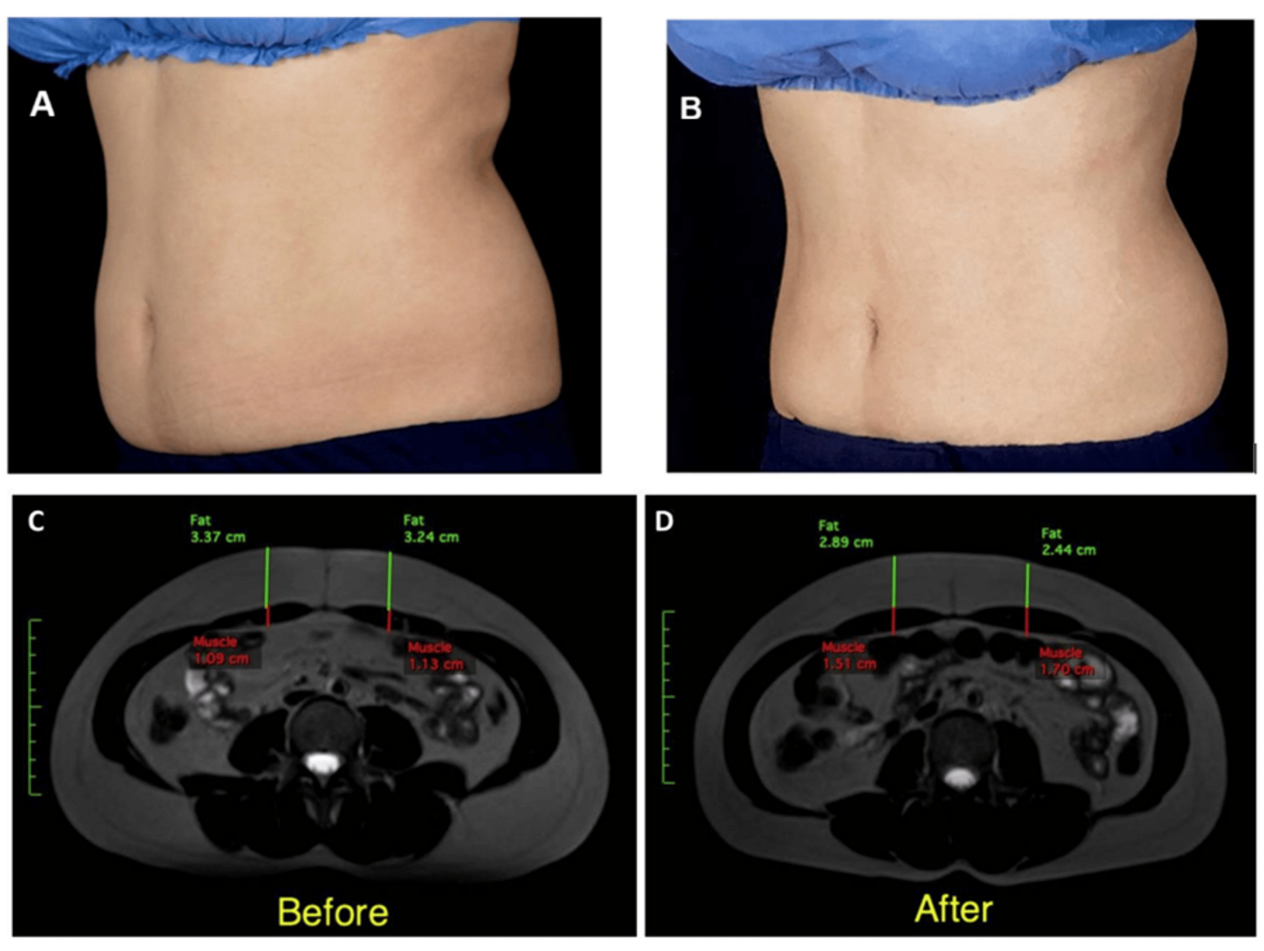
(A) Abdominal photograph of patient No. 9 before treatment, (B) After the treatment, (C) Abdominal MRI Scan of patient No. 9 before treatment, (D) After the treatment

Paired t-tests revealed a significant decrease in waist circumference following treatment (mean reduction: −2.76 ± 1.37 cm; *p* < 0.001). All participants demonstrated a decrease in waist circumference, although the magnitude varied among individuals. The mean weight reduction from baseline to the 10-week follow-up was −0.6 ± 1.35 kg, which was not statistically significant (*p* > 0.05). MRI analysis using paired t-tests demonstrated a significant increase in rectus abdominis thickness from baseline to 10 weeks post-treatment (mean increase: +5.98 ± 1.87 mm; *p* < 0.001), and a significant reduction in abdominal subcutaneous adipose tissue thickness (mean reduction: −4.35 ± 2.80 mm; *p* < 0.001). Representative pre- and post-treatment MRI scans are shown in Figure 1.

At study completion, all participants reported subjective improvement in abdominal muscular strength following the treatment course, and the responses from the patient-reported questionnaires indicated high patient satisfaction (Table 1). Treatment was well tolerated, and no serious adverse events occurred.

**Table 1:** Results of the patient reported questionnaire for each participant

Patient No.	Sex	Patient’s perceived muscle contraction intensity during treatment (1-5 scale)	Patient’s perceived improvement in muscular strength (1-5 scale)	Patient satisfaction with body shape (1-5 scale)
1	Female	4	3	4
2	Female	4	5	5
3	Male	5	4	4
4	Male	5	5	5
5	Male	3	3	4
6	Female	3	3	3
7	Female	4	5	4
8	Male	5	5	5
9	Female	4	4	5
10	Female	4	4	4
	Mean	4.1	4.1	4.3
	Standard Deviation	0.73	0.87	0.67

## Discussion

This pilot case series provides preliminary evidence that FMS applied to the abdominal region can increase rectus abdominis muscle thickness and reduce subcutaneous adipose tissue. All participants demonstrated measurable improvements in waist circumference, and MRI confirmed significant increases in rectus abdominis muscle size accompanied by reductions in abdominal fat thickness. In addition, participants reported improved abdominal muscle strength and high satisfaction with body contour outcomes. Importantly, the intervention was well tolerated, and no serious adverse events were observed.

Our findings are consistent with the proposed mechanism of FMS, whereby high-intensity magnetic fields induce supramaximal muscle contractions that cannot be achieved through voluntary exercise [11-13]. These contractions stimulate deep muscle fibers, potentially promoting hypertrophy and increasing metabolic activity in surrounding adipose tissue [14]. Previous reports have suggested similar improvements in muscle definition and fat reduction with electromagnetic stimulation devices, but much of the existing evidence is limited to small scale observational studies [15-18]. By incorporating MRI-based measurements, this study adds objective imaging evidence to support the efficacy of FMS for abdominal remodelling.

While the reduction in waist circumference (mean −2.76 cm) and abdominal fat thickness (mean −4.35 mm) may appear modest, it is noteworthy that these changes occurred after only 10 sessions, without lifestyle modification or weight reduction. The average weight loss across participants was not statistically significant, suggesting that improvements were localized rather than attributable to overall weight change. This highlights the potential role of FMS as a complementary aesthetic intervention for individuals seeking muscle toning and body contouring beyond what can be achieved with conventional exercise or diet alone [19,20].

Patient-reported outcomes were also favorable. Most participants rated muscle contraction intensity as high, perceived improvements in muscle strength, and expressed satisfaction with abdominal appearance. The absence of major adverse events reinforces the safety of this approach.

Nevertheless, several limitations must be acknowledged. First, the sample size was small, limiting the generalizability of the findings. Second, the absence of a control or sham-treated group prevents exclusion of placebo effects, measurement variability, or the influence of unreported lifestyle changes. Third, the follow-up period was limited to 10 weeks after treatment; longer-term durability of the observed effects remains uncertain. Moreover, the higher cost might hinder application of FMS in clinical settings. Finally, participants were healthy volunteers, so clinical applicability to broader populations requires further investigation.

Future studies should therefore incorporate randomized controlled designs with larger sample sizes, longer follow-up intervals, and standardized sham protocols to validate these preliminary findings. Objective outcomes such as MRI and ultrasound imaging, combined with functional assessments of core strength, will be essential to establish the clinical value of FMS as an aesthetic and therapeutic modality.

## Conclusions

In this prospective pilot case series, FMS of the abdomen was associated with increased rectus abdominis muscle thickness, reduced subcutaneous adipose tissue, and decreased waist circumference, without significant changes in overall body weight. Participants reported high satisfaction with treatment outcomes, and the procedure was well tolerated. These findings support the feasibility and potential efficacy of FMS for abdominal remodeling. However, randomized controlled trials with larger cohorts are warranted to confirm these results and to better define the clinical role of FMS in both aesthetic and functional medicine.

## Figures and Tables

**Figure 2:**
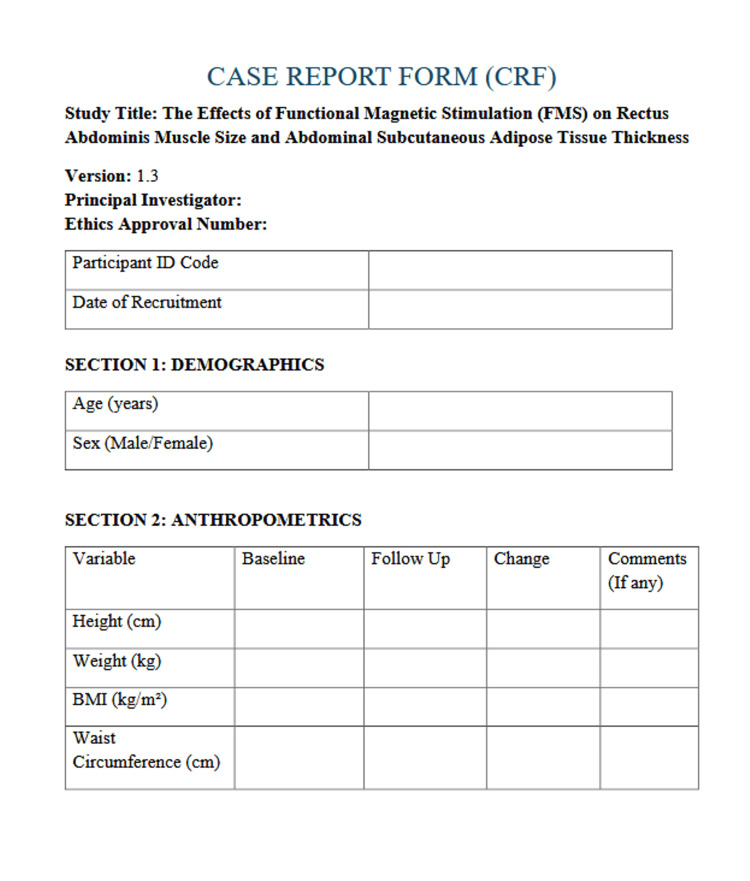
Case Report Form, page 1

**Figure 3:**
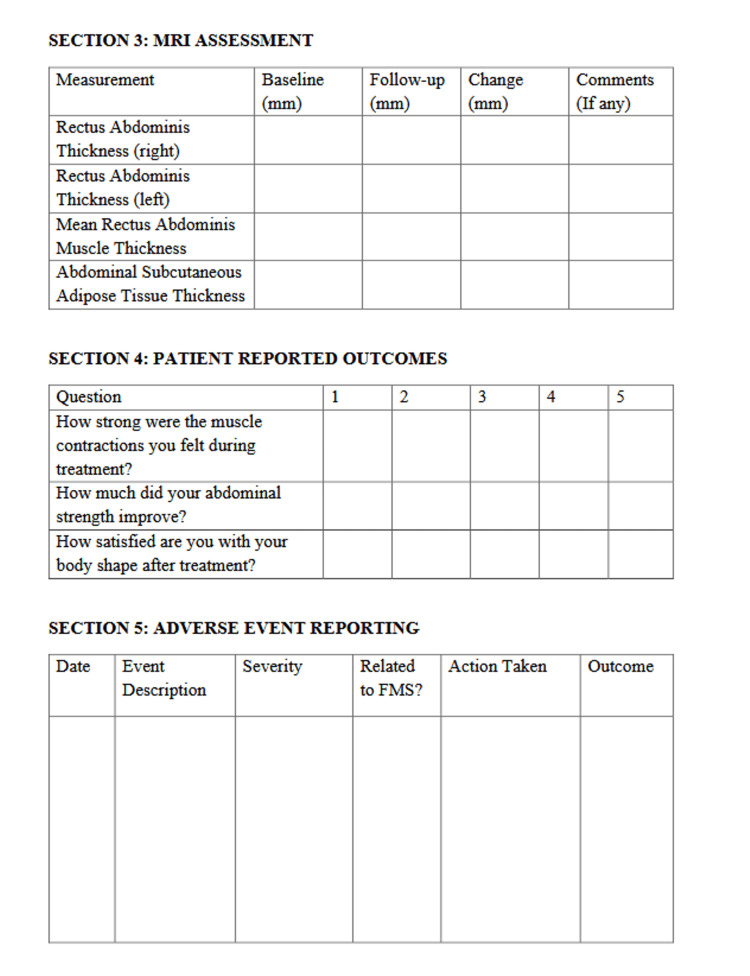
Case Report Form, page 2
